# Complex diagnosis and management of metastatic esophagogastric junction adenocarcinoma with colonic and meningeal involvement

**DOI:** 10.31744/einstein_journal/2025RC1460

**Published:** 2025-10-20

**Authors:** Isadora Yasbick Spricido, Tomás Costa Bignoto, Yuri Greb Vazquez, Pedro Henrique Zavarize de Moraes

**Affiliations:** 1 Hospital Israelita Albert Einstein Department of Medical Oncology São Paulo SP Brazil Department of Medical Oncology. Hospital Israelita Albert Einstein, São Paulo, SP, Brazil.

**Keywords:** Esophagogastric junction, Colon, transverse, Adenocarcinoma, Meningeal carcinomatosis, Esophageal neoplasms, Colon neoplasms, Neoplasm metastasis, Disease progression

## Abstract

This report presents a case of a 59-year-old male diagnosed with metastatic esophagogastric junction adenocarcinoma, who presented with mild dysphagia and vertigo. Brain magnetic resonance imaging initially showed no abnormalities. Upper gastrointestinal endoscopy revealed a vegetative lesion in the distal esophagus, and concurrent colonoscopy identified a laterally-spreading tumor in the transverse colon. Immunohistochemistry confirmed a poorly differentiated adenocarcinoma, suggesting an upper gastrointestinal origin. Further investigation of the worsening neurological symptoms led to the identification of leptomeningeal carcinomatosis via cerebrospinal fluid analysis. Somatic genetic testing detected TP53 mutations and CDK6 amplification; however, no germline mutations were identified. The patient received first-line treatment with 5-fluorouracil, leucovorin, oxaliplatin, and nivolumab, followed by whole-brain radiotherapy due to rapid progression of the leptomeningeal disease. Despite aggressive treatment, the patient experienced multiple complications, including pulmonary embolism and seizures, and died from disease progression after six cycles of therapy. This case highlights the diagnostic challenges and poor prognosis of metastatic esophagogastric junction adenocarcinomas with de novo meningeal and colonic involvement and underscores the importance of early diagnosis and comprehensive evaluation of atypical presentations to optimize patient management.

## INTRODUCTION

Adenocarcinoma of the esophagogastric junction (EGJ) is a significant oncological challenge due to its high incidence and mortality, complex etiology, diverse presentation spectrum, and complicated management.^(1)^

Leptomeningeal carcinomatosis (LC) is a rare complication of malignant diseases, with gastrointestinal tumors accounting for a minority of cases.^(2–4)^ Furthermore, the initial symptoms are commonly nonspecific, and a diagnosis based on neuraxis magnetic resonance imaging (MRI) and cerebrospinal fluid (CSF) evaluation is often challenging.^(3)^ Once diagnosed, the prognosis remains poor, with a median overall survival ranging from 2–6 months.^(5,6)^

This report presents a case of a male patient who initially presented with mild dysphagia and vertigo. Investigation led to the diagnosis of EGJ adenocarcinoma that had metastasized to the colon and meninges. This case report highlights a rare initial presentation and contributes valuable insight to an area with limited existing knowledge.

## CASE REPORT

**Figure 1:**
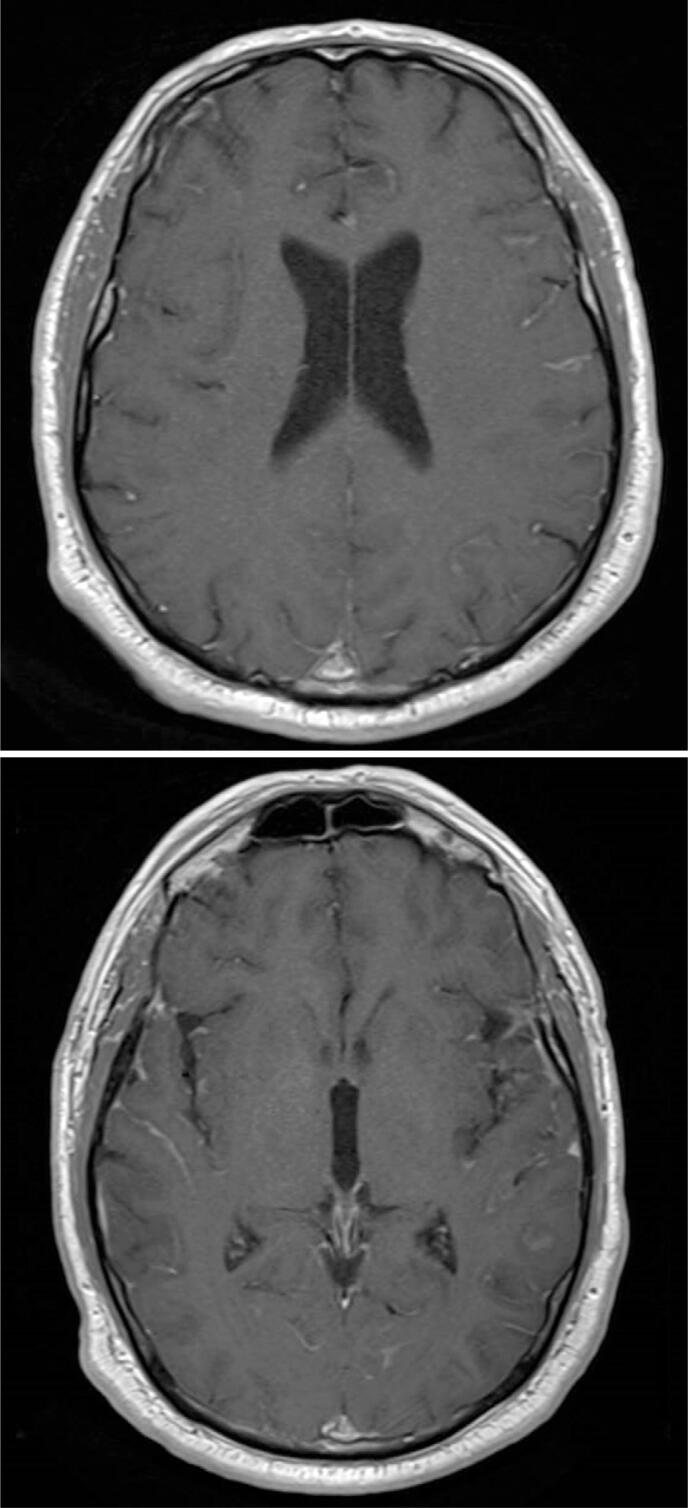
Signs of leptomeningeal contrast enhancement were observed in both supra and infratentorial compartments, extending to several cranial nerves. These findings are consistent with leptomeningeal carcinomatosis

A 59-year-old male patient with a medical history of hypertension, type 2 diabetes, obesity, dyslipidemia, and esophagitis developed mild dysphagia and vertigo 2 months prior to his presentation. An initial brain evaluation using MRI failed to elucidate the cause of the vertiginous symptoms. However, upper gastrointestinal endoscopy revealed a vegetative lesion in the distal esophagus. Colonoscopy was performed concurrently and revealed a laterally-spreading tumor in the transverse colon. Both lesions were biopsied, and pathology confirmed poorly differentiated adenocarcinoma, with immunohistochemistry suggesting an upper gastrointestinal tract (Tables 1 and 2), HER2-negative (score, 0) lesion with proficient mismatch repair (pMMR). A few days later, the patient was admitted with worsening vertigo, confusion, and severe holocranial headaches. The patient underwent a second MRI of the brain, which showed leptomeningeal enhancement (Figure 1). Cerebrospinal fluid analysis revealed neoplastic cells. The patient underwent systemic staging with PET-CT, which revealed no evidence of disease at other sites. Somatic genetic analysis revealed TP53 mutations and CDK6 amplification. Germline testing did not reveal any pathological mutations.

**Table 1:** Immunohistochemical analysis of biopsy from the esophageal lesion

Markers	Antibody/Clone	Results
MLH1	ES05	Positive (preserved expression)
MSH2	FE11	Positive (preserved expression)
MSH6	EP49	Positive (preserved expression)
PMS2	EP51	Positive (preserved expression)
HER2	Policlonal	Negative (score, 0)
CK7	OV-TL12-30	Focally positive
CK20	Ks20,8	Focally positive
SATB2	EP281	Focally positive
CDX2	DAK-CDX2	Positive
MUC1	H23	Positive
MUC2	CCP58	Focally positive
MUC5AC	CLH-2	Negative
TTF1	8G7G3/1	Negative
PSA	Policlonal	Negative
Chromogranin	LK2H10	Positive in rare cells
Synaptophysin	DAK-SYNAP	Positive in rare cells
P40	Policlonal	Negative

**Table 2:** Immunohistochemical analysis of biopsy from the colonic lesion

Markers	Antibody/Clone	Results
Pan-cytokeratin AE1/AE3	AE-1/AE-3	Positive
TTF1	8G7G3/1	Negative
CDX2	DAK-CDX2	Positive
CK20	Ks20,8	Focally positive
SATB2	EP281	Weak and focally positive
PSA	Policlonal	Negative

The patient was diagnosed with EGJ adenocarcinoma with colonic and meningeal metastases. First-line treatment with 5-fluorouracil, leucovorin, oxaliplatin, and nivolumab was initiated.

After one treatment cycle, the patient exhibited increased headache severity and signs of intracranial hypertension, necessitating the placement of a ventriculoperitoneal shunt. Subsequently, the patient underwent whole-brain radiotherapy (total dose: 30Gy). The patient underwent six cycles of this chemoimmunotherapy regimen; however, he experienced multiple clinical complications, including pulmonary embolism, seizures, and bronchoaspiration pneumonia, leading to death.

This case study was approved by the Research Ethics Committee of *Hospital Israelita Albert Einstein* (CAAE: 82925224.4.0000.0071; # 7.062.984). The requirement of informed consent was waived as the patient died and the study did not generate any information that would modify the care of his descendants.

## DISCUSSION

Leptomeningeal carcinomatosis is a rare complication of patients with cancer, occurs in 5–15% of malignant diseases, and is typically more associated with lymphoma, leukemia, melanoma, and lung tumors.^(2,3)^ In esophageal and gastric cancers, the prevalence of LC remains between 0.16–0.69%, with only a few reported cases.^(4)^ The prognosis of this clinical entity is unfavorable, and overall survival ranges from weeks to a few months.^(5,6)^

The clinical presentation of LC is nonspecific, manifesting as headache, vertigo, focal neurological deficits, and signs of intracranial hypertension.^(7)^ In addition to clinical suspicion, diagnosis relies on radiographic findings of neuraxis imaging or the detection of malignant cells in the CSF.^(7)^

Diagnosis of LC is often challenging; in patients with suggestive symptoms, neuraxis MRI is recommended, as it may reveal irregular and nodular leptomeningeal enhancement and assist in the diagnostic process.^(8)^ However, the sensitivity of MRI scanning with gadolinium contrast for diagnosing LC is only about 70%, and a negative result does not exclude the diagnosis.^(9)^

Cerebrospinal fluid analysis via lumbar puncture may lead to a conclusive diagnosis. First, a CSF volume of at least 10ml must be obtained. If the initial sample is negative for malignancy, but the clinical presentation is highly suggestive of LC, lumbar puncture should be repeated. Although CSF cytology yields positive results in only 50–70% of patients, it becomes positive in over 90% of patients after undergoing three high-volume lumbar punctures.^(3,6,9)^

The management of patients with LC should be guided by the recommendations of a multidisciplinary tumor board. In addition to standard systemic therapy tailored to each histological subtype, other approaches could be beneficial, including intrathecal pharmacotherapy and radiotherapy, which often includes whole brain radiotherapy (WBRT) for extensive or symptomatic disease.^(5,10)^

The current first-line treatment for esophagogastric junction adenocarcinoma without HER-2 overexpression or microsatellite instability is a combination of chemotherapy and immunotherapy.^(11–14)^ In Brazil, the use of nivolumab is independent of PD-L1 expression.^(15)^

## CONCLUSION

This report presents a rare case of leptomeningeal carcinomatosis, which typically presents at a later stage of malignant disease. Its primary objective is to highlight the importance of early clinical suspicion and accurate diagnosis to optimize treatment strategies and ultimately improve patient outcomes.

## References

[1] Bray F, Laversanne M, Sung H, Ferlay J, Siegel RL, Soerjomataram I (2024). Global cancer statistics 2022: GLOBOCAN estimates of incidence and mortality worldwide for 36 cancers in 185 countries. CA Cancer J Clin.

[2] Marenco-Hillembrand L, Bamimore MA, Rosado-Philippi J, Perdikis B, Abarbanel DN, Quinones-Hinojosa A (2023). The evolving landscape of leptomeningeal cancer from solid tumors: a systematic review of clinical trials. Cancers (Basel).

[3] Wang N, Bertalan MS, Brastianos PK (2018). Leptomeningeal metastasis from systemic cancer: Review and update on management. Cancer.

[4] Giglio P, Weinberg JS, Forman AD, Wolff R, Groves MD (2005). Neoplastic meningitis in patients with adenocarcinoma of the gastrointestinal tract. Cancer.

[5] Le Rhun E, Weller M, Brandsma D, Van den Bent M, de Azambuja E, Henriksson R, Boulanger T, Peters S, Watts C, Wick W, Wesseling P, Rudà R, Preusser M, EANO Executive Board and ESMO Guidelines Committee (2017). EANO-ESMO Clinical Practice Guidelines for diagnosis, treatment and follow-up of patients with leptomeningeal metastasis from solid tumours. Ann Oncol.

[6] Nayar G, Ejikeme T, Chongsathidkiet P, Elsamadicy AA, Blackwell KL, Clarke JM (2017). Leptomeningeal disease: current diagnostic and therapeutic strategies. Oncotarget.

[7] Le Rhun E, Taillibert S, Chamberlain MC (2013). Carcinomatous meningitis: Leptomeningeal metastases in solid tumors. Surg Neurol Int.

[8] Collie DA, Brush JP, Lammie GA, Grant R, Kunkler I, Leonard R (1999). Imaging features of leptomeningeal metastases. Clin Radiol.

[9] Sindhu KK, Chang S, Liu J, Bakst RL, Dharmarajan KV (2019). In a Patient With Cancer, Not All That Enhances Is Leptomeningeal Carcinomatosis. J Oncol Pract.

[10] Le Rhun E, Preusser M, van den Bent M, Andratschke N, Weller M (2019). How we treat patients with leptomeningeal metastases. ESMO Open.

[11] National Comprehensive Cancer Network (NCCN) (2025). Esophageal and esophagogastric junction cancers. NCCN Clinical Practice Guidelines in Oncology (NCCN Guidelines). Version 1.2024.

[12] Janjigian YY, Shitara K, Moehler M, Garrido M, Salman P, Shen L (2021). First-line nivolumab plus chemotherapy versus chemotherapy alone for advanced gastric, gastro-oesophageal junction, and oesophageal adenocarcinoma (CheckMate 649): a randomised, open-label, phase 3 trial. Lancet.

[13] Sun JM, Shen L, Shah MA, Enzinger P, Adenis A, Doi T, Kojima T, Metges JP, Li Z, Kim SB, Cho BC, Mansoor W, Li SH, Sunpaweravong P, Maqueda MA, Goekkurt E, Hara H, Antunes L, Fountzilas C, Tsuji A, Oliden VC, Liu Q, Shah S, Bhagia P, Kato K, KEYNOTE-590 Investigators (2021). Pembrolizumab plus chemotherapy versus chemotherapy alone for first-line treatment of advanced oesophageal cancer (KEYNOTE-590): a randomised, placebo-controlled, phase 3 study. Lancet.

[14] Rha SY, Oh DY, Yañez P, Bai Y, Ryu MH, Lee J, Rivera F, Alves GV, Garrido M, Shiu KK, Fernández MG, Li J, Lowery MA, Çil T, Cruz FM, Qin S, Luo S, Pan H, Wainberg ZA, Yin L, Bordia S, Bhagia P, Wyrwicz LS, KEYNOTE-859 investigators (2023). Pembrolizumab plus chemotherapy versus placebo plus chemotherapy for HER2-negative advanced gastric cancer (KEYNOTE-859): a multicentre, randomised, double-blind, phase 3 trial. Lancet Oncol.

[15] Brasil (2022). Ministério da Saúde. Agência Nacional de Vigilância Sanitária (ANVISA). Opdivo® (Nivolumabe): nova indicação. Diário Oficial da União. 2022 16 de maio.

